# Metabolic engineering of *Escherichia coli* for high-yield dopamine production via optimized fermentation strategies

**DOI:** 10.1128/aem.00159-25

**Published:** 2025-05-08

**Authors:** Xu Li, Yanghao Liu, Ling Ma, Wenjing Jiang, Tangen Shi, Lanxiao Li, Changgeng Li, Zhichao Chen, Xiaoguang Fan, Qingyang Xu

**Affiliations:** 1 College of Biotechnology, Tianjin University of Science and Technology 534786, Tianjin, China; 2 Key Laboratory of Industrial Fermentation Microbiology of the Ministry of Education, Tianjin University of Science and Technology 562586 , Tianjin, China; Kyoto University, Kyoto, Japan

**Keywords:** dopamine, *Escherichia coli*, metabolic engineering

## Abstract

Dopamine (DA) is a high-value metabolic product; however, its biosynthesis has multiple limitations due to metabolic regulation and fermentation strategies. This study aimed to construct a high-yield dopamine-producing *Escherichia coli* strain devoid of plasmids and defects using *E. coli* W3110 as the chassis strain. We constitutively expressed the *DmDdC* gene from *Drosophila melanogaster* in *E. coli*, which was combined with the *hpaBC* gene from *E. coli* BL21 (DE3), successfully constructed a dopamine biosynthesis module, and achieved preliminary dopamine synthesis in *E. coli*. By optimizing the promoters of the key enzyme genes, we achieved a coordinated balance between the generation and utilization of intermediate metabolites. Subsequently, we used metabolic engineering strategies, such as increasing the carbon flux through the dopamine synthesis pathway, elevating the gene copy number of key enzymes, and constructing an FADH_2_-NADH supply module to create a high-yield strain, DA-29. In this study, a two-stage pH fermentation strategy was developed to enhance fermentation. The first stage ensures the normal growth of the strain, whereas the second stage reduces dopamine degradation by maintaining a low pH. Finally, using a combined Fe^2+^ and ascorbic acid feeding strategy, we obtained 22.58 g/L of dopamine in a 5 L bioreactor, demonstrating that the constructed strain DA-29 possesses high dopamine production capacity, providing strong support for the industrial-scale dopamine production.

**IMPORTANCE:**

In this study, we developed a plasmid-free, defect-free *Escherichia coli* strain with high dopamine production. We further optimized the fermentation process for this strain by applying the dual-stage pH fermentation strategy developed in this research, combined with an Fe²⁺-ascorbic acid co-feeding strategy. This approach significantly increased dopamine yield and addressed the issue of dopamine oxidation during fermentation. The yield reached 22.58 g/L, marking the highest known yield to date and laying a solid foundation for future scale-up production. This research explores the metabolic pathway of dopamine and the efficient fermentation methods for its production, providing a novel fermentation strategy. It offers new insights into microbial production of aromatic amino acid derivatives, advancing research in this field.

## INTRODUCTION

Dopamine (3,4-dihydroxyphenethylamine, DA) is a high-value metabolic product involved in neurophysiological activities such as learning and motivation (1, 2). As a neurotransmitter, DA serves as a precursor to norepinephrine and epinephrine and plays an important role in regulating a range of physiological functions in the body. It is frequently used in clinical practice to treat various types of shock (3). Under specific conditions, DA undergoes oxidation and self-polymerization to form polydopamine (PDA), widely used in biomaterials (4–6). Owing to its importance in the pharmaceutical and biomaterial fields, the demand for DA continues to increase. The synthesis of DA relies primarily on chemical methods that are complex, expensive, and toxic raw materials (7). Therefore, there is an urgent need to develop sustainable, safe, and cost-effective methods for synthesizing DA.

In the biosynthesis of DA, tyrosine (Tyr) is typically used as a substrate, which is hydroxylated to form L-DOPA and subsequently decarboxylated to produce DA. 4-hydroxyphenylacetic acid 3-monooxygenase (encoded by the *hpaBC* gene) comprises monooxygenase HpaB and flavin reductase HpaC. HpaB catalyzes the ortho-hydroxylation of 4-HPA analogs (phenol, para-cresol, and Tyr) in *E. coli*, whereas HpaC acts as a coupling factor, providing FADH_2_ for HpaB hydroxylation (8–10). Wei et al. successfully constructed an *E. coli* strain for L-DOPA synthesis by deleting transcriptional regulatory factors, modifying the glucose transport system, and knocking out branched pathways. They further enhanced L-DOPA production using multiplex automated genome engineering (MAGE) technology and achieved a titer of 8.67 g/L (11). Eric et al. achieved an acid yield of 25.53 g/L in a 5 L bioreactor by regulating the carbon flux in *E. coli* BL21 (DE3) and performing directed evolution to optimize the activity of HpaB (12). These results suggest that the use of *hpaBC* in L-DOPA synthesis has great potential for practical applications.

Recent studies have also reported a 5′-phosphopyridoxal-dependent enzyme, dopamine decarboxylase (DDC, EC 4.1.1.28), that catalyzes the conversion of L-DOPA to DA (13). Researchers are currently exploring biological approaches for DA synthesis. Gao et al. expressed the *DDC* gene from *Harmonia axyridis* in *E. coli* BL21(DE3) and used enzyme catalysis to synthesize DA under anaerobic conditions using L-DOPA as the substrate, yielding 21.99 g/L (14). Subsequently, researchers overexpressed the transcriptional protein AroP and optimized the process conditions, further increasing yield (15). Das et al. expressed the *hpaBC* genes from *E. coli* BL21(DE3) in *E. coli* DH5α and introduced the *DDC* gene (*ssDdC*) from *Sus scrofa*. They added cofactors as supplemental components to the culture medium and used Tyr as the substrate for fermentation, obtaining 27 mg/L of DA after 24 h in shake flask cultures (16). Nakagawa et al. used an *E. coli* BL21(DE3) strain with the TyrR repressor factor knocked out and expressed key genes in the DA synthesis pathway (*tyrA^fbr^*, *aroG^fbr^*, *tktA*, *ppsA*, *Rstyr*, and *dodc*) using a dual-plasmid system. The strain can produce DA at 2.15 g/L, with a molar conversion rate of 3.8% (17).

The DA biosynthesis pathway involves complex metabolic flux distributions and intricate fermentation conditions. Thus, the large-scale industrial production of DA is yet to be achieved. The main challenges include (i) extensive metabolic diversion in *E. coli*, causing significant carbon loss; (ii) the presence of multiple feedback inhibition sites in the DA metabolic pathway; (iii) difficulty in effectively supplementing cofactors (FADH_2_ and NADH) during fermentation, and (iv) the susceptibility of DA to oxidation. Although some studies have explored the biosynthesis of DA, most have focused on the accumulation of precursor substances (such as tyrosine), and the yields of engineered strains are typically low. Therefore, advancing the current modification approaches and constructing high-yield DA-producing strains is imperative.

**Fig 1:**
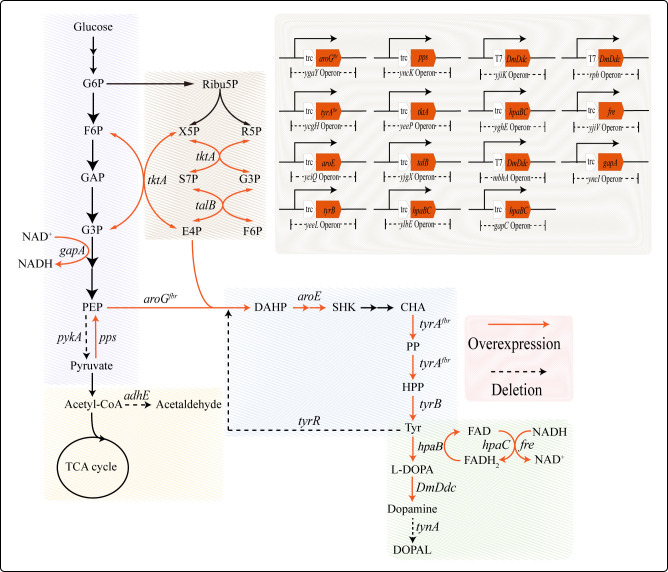
Dopamine biosynthesis pathway. Abbreviations: G6P, glucose-6-phosphate; F6P, P fructose-6-phosphate; G3P, glyceraldehyde 3-phosphate; PEP, phosphoenolpyruvate; Ribu5P, ribulose-5-phosphate; X5P, xylulose-5-phosphate; R5P, ribose-5-phosphate; S7P, Sedoheptulose 7-phosphate; E4P, erythrose 4-phosphate; DAHP, 3-deoxy-d-arabino-heptulosonate-7-phosphate synthase; SHK, Shikimate; CHA, chorismate; PP, Prephenate; HPP, 4-Hydroxyphenylpyruvate; Tyr, L-Tyrosine; L-DOPA, 3,4-Dihydroxy-L-phenylalanine; DOPAL, 3,4-Dihydroxyphenylacetaldehyde.

In this study, we developed a DA-producing strain using *E. coli* W3110 as the chassis strain. The DA yield was significantly improved by optimizing the synthesis pathway and developing corresponding fermentation technologies. We successfully constructed a high-yield strain, DA-29, using strategies such as designing DA synthesis elements, optimizing key enzyme promoters, modulating metabolic flux, introducing multi-copy expression of key enzymes, and establishing cofactor supply modules, as shown in Fig. 1. Based on the characteristics of this strain, we further optimized the fermentation process by developing a dual-stage pH-controlled fermentation strategy and designing a mixed feeding approach involving Fe^2+^ and ascorbic acid, which significantly enhanced the fermentation potential of the strain. Using the fermentation technology developed in this study, strain DA-29 achieved a DA yield of 22.58 g/L and a carbon molar conversion rate of 3.37% in a 5 L bioreactor. This study presents a novel strategy for microbial DA synthesis and establishes a cost-effective DA fermentation process that provides a solid foundation for industrial-scale production.

## RESULTS

### Preliminary synthesis of DA in *E. coli*

Owing to its well-defined genetic background and easily controllable metabolic characteristics, the *E. coli* W3110 strain is commonly used as a host for the molecular manipulation and construction of various recombinant strains. Therefore, *E. coli* W3110 was selected as the chassis strain in this study. The knockout of the product degradation pathway is a commonly used strategy in metabolic engineering to promote product accumulation. In *E. coli*, tyramine oxidase (TynA) can oxidize DA to 3,4-dihydroxyphenylacetaldehyde, releasing equivalent amounts of H_2_O_2_ and NH_3_ (18, 19). Therefore, we knocked out *tynA* in *E. coli* W3110 and integrated the *T7 RNA polymerase* gene driven by the xylose promoter at the *LacI* locus to obtain strain DA-1. To synthesize the intermediate product L-DOPA, we introduced the *hpaBC* genes from *E. coli* BL21(DE3) into the DA-1 strain and controlled the expression of the related genes using the lac promoter, resulting in the strain DA-1–1.

**Fig 2:**
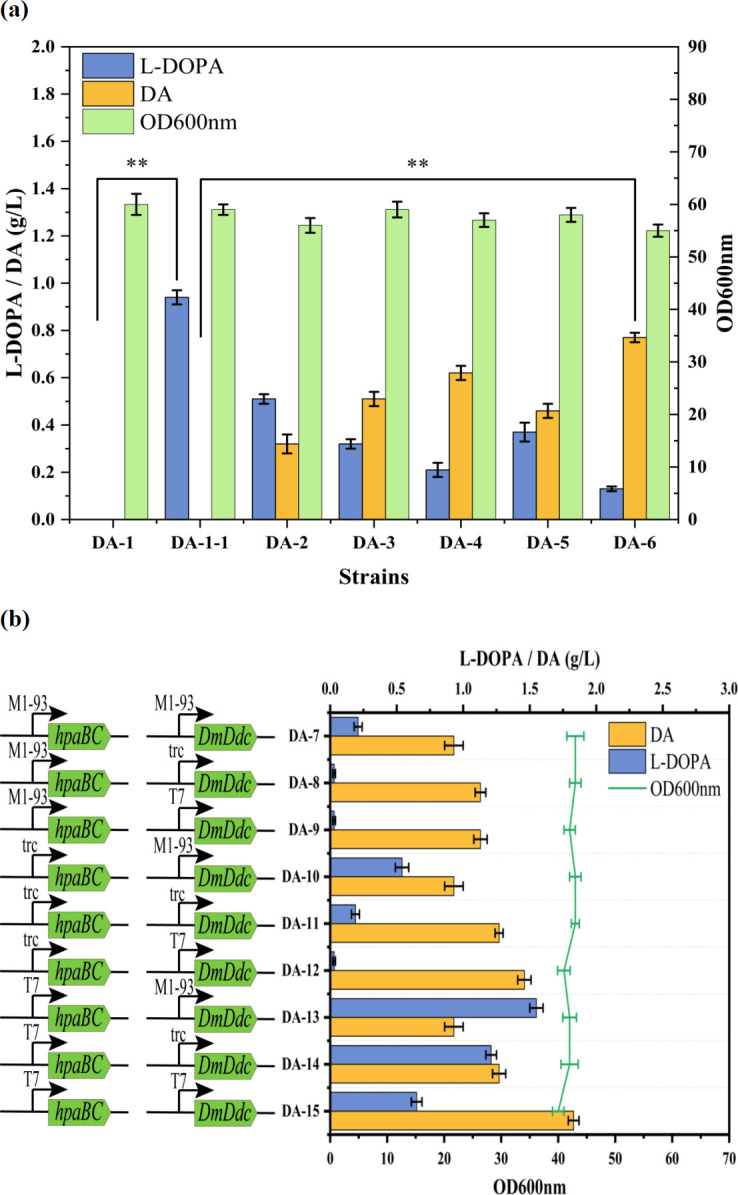
Construction of the dopamine synthesis module and promoter optimization. (**a**) Establishment of the L-DOPA synthesis module in the DA-1 strain and evaluation of the effects of different DDC genes on dopamine synthesis, with partial fermentation results displayed. (**b**) Comparison of dopamine production and L-DOPA accumulation levels in different strains by regulating hpaBC and DmDdc expression using different promoter combinations (M1-93, trc, T7), along with OD600 nm measurements.

We screened five efficient decarboxylase (*DDC*) genes to improve the ability of this strain to synthesize DA. In this study, we selected five *DDC* genes, HaDdc from *Harmonia axyridis* (14), SsDdc from *Sus scrofa* (16), HsDdc from *Homo sapiens* (20), CfDdc from *Chlamys farreri* (21), and DmDdc from *Drosophila melanogaster* (22), to verify their expression efficiency in *E. coli*. These five *DDC* genes were integrated into the DA-1–1 strain, with the expression of the relevant genes controlled by the lac promoter. Five recombinant strains were identified. Next, we added Tyr to the five recombinant strains for shake-flask fermentation for 24 h and quantified the DA yield in the samples. The results showed that after the introduction of the hpaBC genes, L-DOPA appeared in the DA-1–1 strain with a titer of 0.94 g/L. We also observed significant differences in DA production among the strains expressing different decarboxylases (Fig. 2a). The DA titers for the five strains were 0.51, 0.62, 0.32, 0.47, and 0.77 g/L, respectively. The DA-6 strain, which contains *DmDdc* from *D. melanogaster*, showed the highest DA production.

### Optimization of key enzyme promoters

Promoters are cis-regulatory elements that govern gene expression and are critical for regulating gene transcription. The strength of a promoter directly affects the level of gene expression. In metabolic engineering pathway optimization, the expression of target genes must be carefully regulated according to the strength of the different promoters to achieve a balance between the production and utilization of intermediate metabolites, thereby enhancing the synthesis efficiency of the desired products. To maintain a low level of L-DOPA during the synthesis process and maximize DA production, we selected three promoters with different transcriptional strengths (T7, trc, and M1-93) to regulate the expression of the *hpaBC* and *DmDdc* genes, with the transcriptional strength decreasing in the order T7 >trc > M1-93.

In the genome of strain DA-1, different combinations were used to express *hpaBC* and *DmDdc* genes, resulting in nine different recombinant strains (Fig. 2b). When the *hpaBC* gene was regulated by the weakest M1-93 promoter, the DA titer initially increased with the strength of the *DmDdc* promoter and then leveled off. The L-DOPA accumulation in strains DA-8 and DA-9 was low (≤0.03 g/L), possibly owing to insufficient L-DOPA supply. When the *hpaBC* gene was regulated by the moderate-strength trc promoter, the DA titer continuously increased with the strength of the *DmDdc* promoter, reaching 1.47 g/L in strain DA-12, whereas L-DOPA accumulation remained low (≤0.03 g/L). When a strong T7 promoter was used to regulate *hpaBC*, the DA titer increased with increasing strength of the *DmDdc* promoter. In particular, strain DA-15, which expressed both *hpaBC* and *DmDdc* under the control of the T7 promoter, achieved a DA titer of 1.83 g/L, and L-DOPA accumulation increased to 0.65 g/L. Therefore, strain DA-12, which exhibited a high DA titer and low L-DOPA accumulation, was selected. The results demonstrated that this combinatorial strategy significantly increased DA yield compared with strains with a single promoter system. By precisely regulating the expression of key enzymes, the engineered strain improved DA production from 0.77 g/L to 1.47 g/L.

### Relieving repression and feedback inhibition

Although DA can be synthesized by introducing DA expression elements, optimizing the promoters of key enzymes, and supplementing with exogenous Tyr, this approach increases the production burden. It also fails to deliver optimal results, ultimately failing to meet expectations. A complete DA synthesis pathway must be established within bacterial cells to address this issue. Strategies such as employing feedback-resistant enzymes, overexpressing pathway enzymes, and increasing intermediate metabolic flux have effectively enhanced aromatic amino acids (23–27).

**Fig 3:**
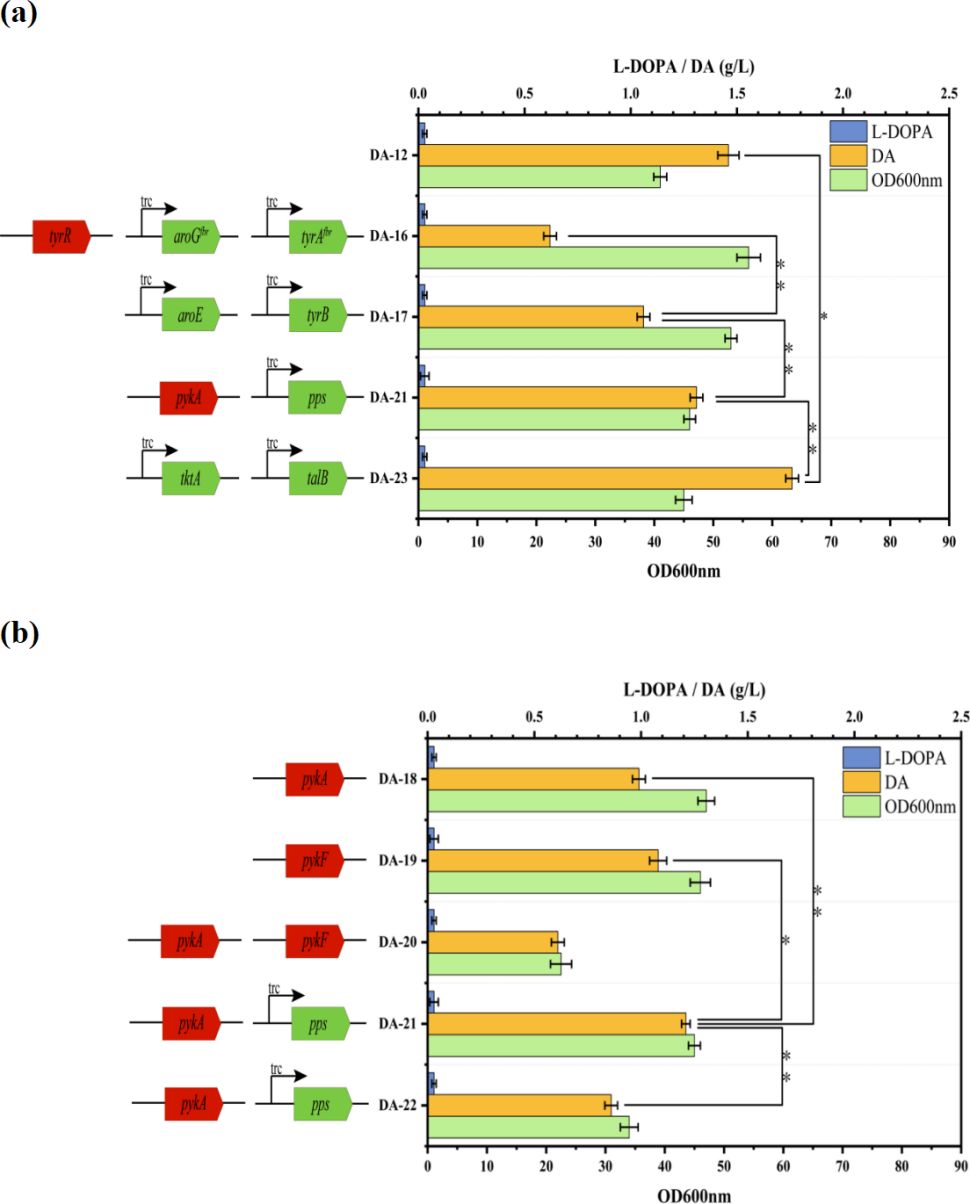
By regulating specific genes to direct the carbon flux toward the target metabolites, the partial fermentation performance of the engineered strains was evaluated. Green arrows and squares represent overexpressed genes, whereas red squares indicate deleted genes. (**a**) The engineered strains produced L-DOPA, dopamine, and OD600 nm while enhancing the carbon flux through the dopamine biosynthesis pathway. (**b**) While balancing the carbon flux between the TCA cycle and the dopamine biosynthesis pathway, the engineered strains produced L-DOPA, dopamine, and OD600 nm.

Repression of metabolic pathways and feedback inhibition of rate-limiting enzymes are key strategies for synthesizing DA. Inactivation of *tyrR* can relieve the inhibition of aromatic amino acid accumulation (28, 29). DAHP synthesis is a key step in the biosynthesis of aromatic amino acids. PEP and E4P are converted to DAHP under the catalysis of 3-deoxy-D-arabino-heptulosonate-7-phosphate (DAHP) synthase. The rate of DAHP production determines the overall pace of the aromatic amino acid synthesis pathway. Therefore, regulating the efficiency of DAHP synthesis in *E. coli* can significantly enhance the synthesis of subsequent metabolic products (30). DAHP synthase is encoded by the *aroG*, *aroF*, and *aroH* genes, with *aroG* accounting for 79% of the total activity, *aroF* for 20%, and *aroH* for 1%. Relieving the feedback inhibition of DAHP synthase is crucial for improving the efficiency of DAHP synthesis. Additionally, studies have shown that chorismate mutase (encoded by the *tyrA* gene) is the rate-limiting enzyme from the branch point acid to the tyrosine pathway, and relieving feedback inhibition of the *tyrA* gene is also significant for DA synthesis (31, 32). Therefore, in strain DA-12, we knocked out the *tyrR* gene and introduced the mutated genes *aroG^fbr^* (D146 N) and *tyrA^fbr^* (M53 I/A354 V) using the trc promoter to control the expression of these genes. As shown in Fig. 3a, the DA-16 strain was generated. By optimizing the expression of pathway enzymes, this strain was able to utilize glucose rather than tyrosine for dopamine synthesis, achieving a titer of 0.57 g/L. This strategy not only enables the strain to synthesize endogenous tyrosine but also facilitates further dopamine production. With further optimization, higher dopamine yields are expected. Additionally, in Fig. 3a, the DA-12 strain is compared, and the results show that endogenous synthesis of tyrosine offers a significant advantage over exogenous addition in dopamine synthesis.

### Enhancing intermediate metabolic flux

In addition, *aroE* in *E. coli* controls the catalysis of DAHP to produce shikimate, a key enzyme in the shikimate pathway. The *tyrB* gene encodes aromatic amino acid aminotransferase that catalyzes the final step of tyrosine biosynthesis (25). To further increase carbon flux in the DA synthesis pathway, we overexpressed *aroE* and *tyrB* in strain DA-16 using the trc promoter, resulting in strain DA-17. Shake flask fermentation tests revealed that the DA titer of strain DA-17 reached 0.89 g/L, a 36.8% increase compared with DA-16.

### Re-engineering central metabolic pathways

*E. coli* utilizes PEP and E4P as substrates, which are condensed by DAHP synthase to generate DAHP, which subsequently enters the shikimate pathway for the biosynthesis of aromatic amino acids. Balancing the carbon flux between the TCA cycle and DA synthesis pathway in optimizing aromatic amino acid production is critical for cell growth and product synthesis (33). To enhance the availability of PEP during DAHP synthesis, it is crucial to redirect PEP without affecting cell growth. PEP is catalyzed to pyruvate by the pyruvate kinases (PykA and PykF). As shown in Fig. 3b, *pykA* and *pykF* were knocked out in DA-17 cells to generate DA-18 and DA-19. DA titers of strains DA-18 and DA-19 were 0.99 g/L and 1.08 g/L, respectively, representing a 26.9% and 38.5% increase compared with DA-17. However, their OD600 nm decreased by 11.3% and 13.2%, respectively, compared with DA-17, indicating that individual knockouts of the *pykA* or *pykF* genes resulted in only a modest increase in DA accumulation. When both *pykA* and *pykF* were simultaneously knocked out, the resulting strain, DA-20, exhibited a dopamine titer of 0.61 g/L with an OD600 nm of 22.5. Pyruvate phosphate dikinase (PPS) converts pyruvate into PEP. In this study, we enhanced the expression of *pps* in strains DA-18 and DA-19 using the trc promoter; DA-21 and DA-22 were generated. Among them, the DA titer in strain DA-21 significantly increased to 1.21 g/L, a 22.2% increase compared with strain DA-18. However, the DA accumulation in strain DA-22 was only 0.86 g/L, a 20.4% decrease compared with DA-19. This finding suggests that excessive direct carbon flux into the DA synthesis pathway weakens the conversion of PEP to pyruvate, thereby reducing the carbon flux to the TCA cycle and affecting normal cell growth. Therefore, we made further modifications based on the best DA titer performance of strain DA-21.

Although we redirected the PEP metabolic pathway toward DA synthesis, the results were not ideal, with only a marginal increase in DA accumulation. This suggests that E4P is a limiting substrate in the DA synthesis pathway. In cases of insufficient E4P supply, increasing the PEP flux did not significantly enhance the DA yield. To address this, we upregulated the expression of genes related to E4P synthesis in strain DA-21. We overexpressed *tktA* and *talB* at the *yeeP* and *yjgX* loci using the trc promoter, resulting in strain DA-23 (34–37). The DA titer in strain DA-23 increased by 29.5% compared to DA-21. In contrast, L-DOPA accumulation remained low (≤0.03 g/L), and cell concentration did not change significantly, indicating that E4P accumulation has an apparent promotive effect on DA synthesis.

### Optimization of gene copy number of key enzymes

**Fig 4:**
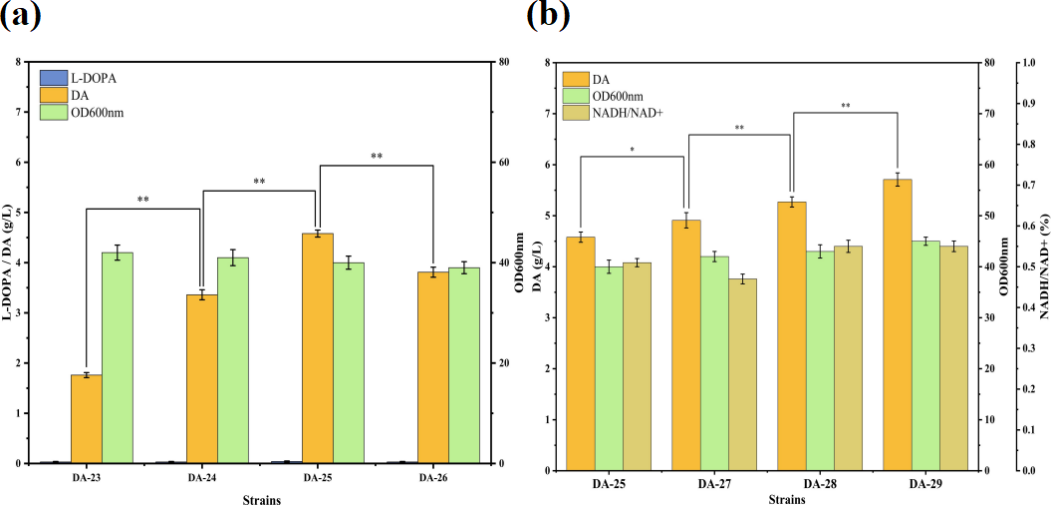
Partial fermentation performance of the engineered strains was evaluated by regulating the expression of relevant genes. (**a**) While optimizing the key enzyme gene copy number, the engineered strains produced L-DOPA, dopamine, and OD600nm. (**b**) During the construction of the FADH2-NADH supply system, the engineered strains produced dopamine, OD600 nm, and the NADH/NAD+ ratio.

To further enhance the DA synthesis capacity, we optimized the expression of *hpaBC* and *DmDdc* genes by introducing double, triple, and quadruple copies in strain DA-23, resulting in strains DA-24, DA-25, and DA-26, with DA-23 serving as a control. As shown in Fig. 4a, the DA titer exhibited an initial increase followed by a decrease as the copy number increased. Strain DA-25 exhibited the highest DA titer, reaching 4.58 g/L, indicating that triple-copy expression was advantageous for DA synthesis. However, an excessive number of gene copies did not further increase DA potency but caused a decrease in yield. Therefore, we selected the three-copy strain DA-25, which had a higher yield, for the next round of modification.

### Construction of the FADH_2_-NADH cofactor supply system

FADH_2_ (flavin adenine dinucleotide in its reduced form) is a derivative of vitamin B2 that acts as a reduced coenzyme in electron transfer and oxidative phosphorylation to generate ATP (38). In *E. coli*, HpaB is a hydroxylase dependent on FADH2 that catalyzes the hydroxylation of tyrosine in the presence of FADH2. Subsequently, hpaC utilizes FAD and NADH to synthesize FADH_2_ within the cell (39). The regeneration rate of FADH_2_ may limit the efficiency of tyrosine hydroxylation, thereby enhancing its regeneration rate of FADH_2_, which is crucial for improving DA synthesis. Flavin reductase, encoded by the *fre* gene in *E. coli* uses flavin as a substrate and consumes NADH to convert FAD to FADH_2_, thereby providing additional FADH_2_ for HpaB (40, 41). In strain DA-25, the expression of the *fre* gene was enhanced using the trc promoter to promote the *in vivo* cycling of FADH_2_, resulting in strain DA-27. The DA titer in strain DA-27 increased by 9.7% compared with DA-25, reaching 4.91 g/L, indicating that overexpression of the *fre* gene effectively promoted the hydroxylation process mediated by HpaBC (Fig. 4b).

Although overexpression of the *fre* gene enhances the hydroxylation efficiency of HpaBC, this process may excessively consume intracellular NADH, which could affect the normal growth and metabolism of the cells. Therefore, regulating NADH levels and maintaining redox stability within the cells is crucial. Lee et al. increased NADH utilization using glycerol as a carbon source and observed a nearly 2-fold increase in L-DOPA yield (42). Strategies for increasing NADH levels include blocking competing NADH pathways and constructing NADH regeneration pathways. Alcohol dehydrogenase (AdhE) catalyzes the reduction of acetyl-CoA to acetaldehyde, which consumes NADH. The *deletion of adhE* in strain DA-27 resulted in strain DA-28. Shake flask fermentation results demonstrated that the DA accumulation and NADH/NAD+ ratio in DA-28 increased by 7.3% (5.27 g/L) and 17.4% (0.54), respectively, compared with that of strain DA-27. Additionally, the *gapA* gene encodes glyceraldehyde-3-phosphate dehydrogenase, which is a major source of NADH during glycolysis. Overexpression of the *gapA* gene can effectively increase intracellular NADH levels. Overexpression of the gapA gene in strain DA-28 resulted in the formation of strain DA-29. Shake flask fermentation results demonstrated that the DA titer in DA-29 reached 5.71 g/L, an 8.4% increase compared with DA-28, whereas the NADH/NAD+ ratio remained unchanged. This finding indicates that enhancing NADH supply indirectly promotes FADH_2_ regeneration and significantly facilitates L-DOPA synthesis, thus improving DA synthesis efficiency.

### Two-stage pH fermentation process

**Fig 5:**
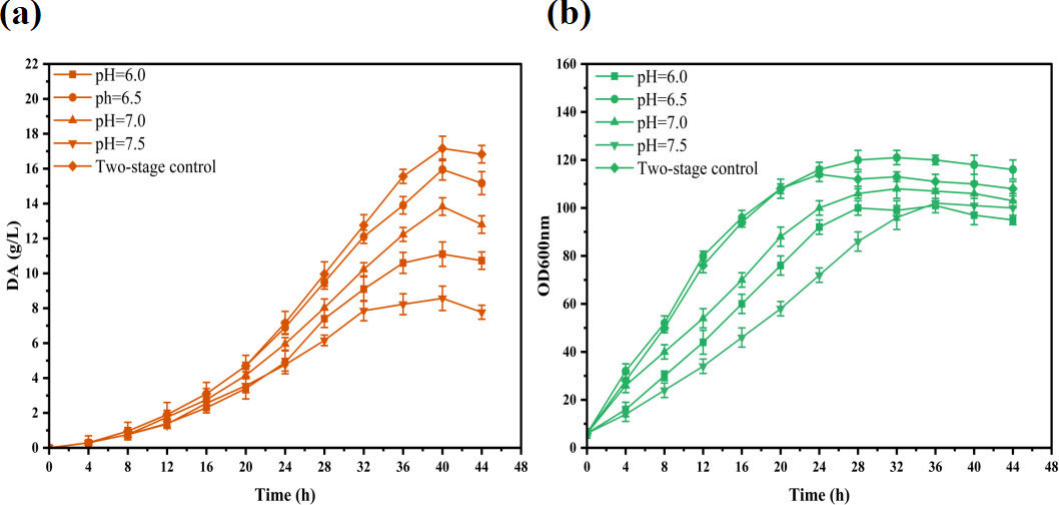
Partial fermentation products of the engineered strains were evaluated by adjusting the pH during the fermentation process. (**a**) The dopamine accumulation in strain DA-29 under different pH conditions. (**b**) The OD600 nm of strain DA-29 under different pH conditions.

During fermentation, we observed that as the pH increased, the color of the fermentation broth gradually turned black, and DA accumulation decreased. Dopamine can be protonated in acidic environments, and protonated DA exhibits high water solubility and stability. Therefore, we hypothesized that increased pH during fermentation may have caused DA degradation, inhibiting its accumulation. To reduce the oxidative degradation of DA, we investigated the effect of different pH conditions on DA synthesis. We selected four pH values (6.0, 6.5, 7.0, and 7.5) and observed their effects on fermentation outcomes (Fig. 5). The fermentation results revealed that the DA yield was lower when the pH exceeded 7.0, possibly because the high pH affected the strain growth, thereby causing some DA degradation. At pH 6.5, the DA titer was the highest, reaching 15.35 g/L, and strain growth was significantly better than that under other pH conditions. At pH 6.0, the DA titer was lower at 11.13 g/L, and the degradation of DA at the end of fermentation was the lowest among all conditions. This observation suggests that while pH 6.0 is not ideal for cell growth, it may be more suitable for preserving DA.

Based on these findings, we explored a two-stage pH regulation fermentation process. The pH was controlled at 6.5 during the initial fermentation stage to support normal cell growth. Once the cells reached the stationary phase (approximately 20 h), the pH was reduced to 6.0 to minimize DA degradation, and a two-stage pH fermentation process was carried out using strain DA-29. The results revealed that the DA titer reached 17.16 g/L, representing an 11.8% increase compared with fermentation at pH 6.5.

### Fe^2+^ and ascorbic acid mixed feeding strategy

Fe^2+^ is a crucial trace element in cell growth and production processes and is involved in various biological metabolic pathways. The active sites of hpaBC require Fe^2+^ to exert catalytic activity. Therefore, maintaining an appropriate concentration of Fe^2+^ in the fermentation environment is an effective strategy for promoting tyrosine hydroxylation and increasing the DA yield (16). However, Fe^2+^ is readily oxidized to Fe^3+^ in the air, which damages cellular macromolecules and leads to cell death (43, 44). Additionally, the hydroxylation reaction involving hpaBC during DA synthesis requires molecular oxygen, and DA is prone to degradation in the presence of oxygen. Therefore, the addition of antioxidants is essential for slowing DA degradation.

**Fig 6:**
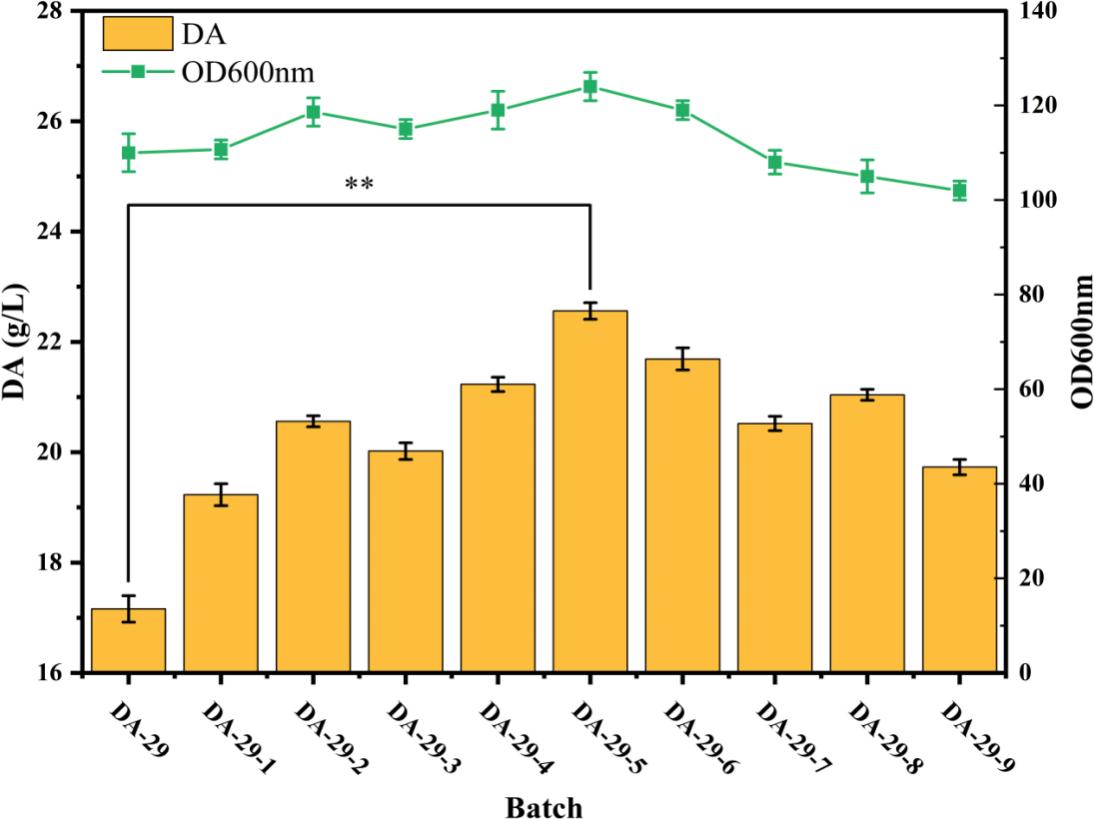
Effect of Fe^2+^ and ascorbic acid ratio on DA accumulation and cell growth.

We developed a mixed feeding strategy involving Fe^2+^ and ascorbic acid to optimize DA production and promote dopamine synthesis. The ratio of Fe^2+^ to ascorbic acid was adjusted in different fermentation batches (Table 1), and the effects of varying the concentrations of these two components on DA accumulation and cell growth were evaluated. As shown in Fig. 6, the experimental results indicated that at a Fe^2+^ concentration of 40 mg/L and an ascorbic acid concentration of 20 mmol/L, the DA concentration reached 22.58 g/L, which exhibited the best production performance. When the Fe^2+^ concentration remained at 40 mg/L, but the ascorbic acid concentration was increased to 30 mmol/L, the DA concentration slightly decreased to 21.69 g/L. This reduction may be attributed to higher ascorbic acid concentration, inhibiting glucose metabolism and limiting DA synthesis (45). Similarly, when the Fe^2+^ concentration was increased to 60 mg/L, and the ascorbic acid concentration was 20 mmol/L, the DA concentration decreased to 21.04 g/L, likely due to the toxic effects of excess Fe^2+^ on the cells. When the Fe^2+^ and ascorbic acid concentrations were increased to 60 and 30 mmol/L, the DA concentration further decreased to 19.93 g/L, suggesting that an excess of both Fe^2+^ and ascorbic acid inhibited DA synthesis. These results underscore the necessity of precisely controlling the concentrations of Fe^2+^ and ascorbic acid, as excessively high concentrations of either component can exert toxic effects on cells, thus reducing DA yield. Therefore, the optimal ratio for batch DA-29–5, with 40 mg/L Fe^2+^ and 20 mmol/L ascorbic acid, was the most effective choice to maximize DA production.

**TABLE 1:** Ratio of Fe^2+^ to ascorbic acid in different fermentation batches

Batch	Fe^2+^ addition	Ascorbic acid addition
DA-29–1	20 mg/L	10 mmol/L
DA-29–2		20 mmol/L
DA-29–3		30 mmol/L
DA-29–4	40 mg/L	10 mmol/L
DA-29–5		20 mmol/L
DA-29–6		30 mmol/L
DA-29–7	60 mg/L	10 mmol/L
DA-29–8		20 mmol/L

## DISCUSSION

DA is a critical intermediate in the biosynthesis of catecholamines such as adrenaline and noradrenaline. As the importance of DA in pharmaceuticals and biomaterials continues to grow, the demand for DA is also rising (46). Compared with gram-positive bacteria, gram-negative bacteria are more advantageous in industrial enzyme production, biofuel synthesis, environmental remediation, and the production of high-value-added compounds. *E. coli*, as one of the simplest model organisms, offers unparalleled advantages in fundamental biological research and biotechnological applications due to its well-characterized background, rapid growth, and ease of manipulation. Additionally, there are no reported studies indicating that endotoxin secretion by *E. coli* affects dopamine synthesis. Therefore, in this study, we designed an efficient dopamine biosynthesis system using *E. coli* as the chassis strain, significantly enhancing DA production. Additionally, we explored the optimization of promoter regulation, cofactor supplementation strategies, and multistage fermentation processes. Our experimental system demonstrated significant improvements in several key aspects compared with previous studies on dopamine biosynthesis in *E. coli*.

Metabolic flux optimization is the key to increasing DA production. Previous studies have improved the efficiency of tyrosine conversion to DA by regulating key genes involved in the biosynthetic pathways. Building on this foundation, we selected efficient DDC enzymes and optimized the promoters of key enzymes, enhancing the reaction efficiency and further boosting DA production. However, single promoter systems may lead to the overexpression of specific genes, disrupting the metabolic balance and negatively affecting the synthesis of the final product. In the present study, we combined the T7, trc, and M1-93 promoters, which regulate the expression of HpaBC and DmDdc to varying extents. The results showed that compared with traditional single-promoter systems, the combination of promoters enabled more precise regulation of key enzyme expression, optimized metabolic flux, and minimized potential metabolic burdens, thereby effectively improving the efficiency of dopamine synthesis. Although this study has made progress in dopamine synthesis through the selection of specific promoters, the impact of promoter regulation and promoter strength on the expression levels of key enzymes suggests that current dopamine production is still limited by metabolic flux imbalance and cellular metabolic burden. Therefore, further screening and optimization of other promoters (such as the inducible promoter ParaBAD or synthetic promoter libraries) to dynamically regulate the expression strength of key genes, balance the distribution of metabolic flux, and reduce the accumulation of toxic intermediates may provide crucial support for the next improvements in the dopamine synthesis pathway. This approach holds significant potential, particularly in enhancing yield, reducing metabolic burden, and achieving feasibility for industrial-scale production. Then, we investigated the effect of multi-copy expression of key enzymes. The study found that the highest DA synthesis occurred with three copies of the expression while further increasing to four copies led to a decline. This phenomenon is likely due to the high expression of exogenous proteins, which increases the biosynthetic burden on the cells. Overexpression may reduce amino acid utilization efficiency, and the accumulation of misfolded proteins and the formation of inclusion bodies could trigger heat shock and nutrient deficiency responses (47).

To enhance the enzyme activity and metabolic efficiency during DA biosynthesis, we constructed an FADH₂-NADH supply system. Optimization of gene expression to regulate FADH II and NADH synthesis experimental results demonstrated that the introduction of this supply system not only enhanced the activity of key enzymes but also facilitated efficient coupling of metabolic pathway steps, maximizing the utilization of cellular resources and avoiding the adverse effects of cofactor depletion on the synthesis process.

Furthermore, optimizing the fermentation process is another critical factor for enhancing DA production. We successfully balanced cell growth and metabolic product synthesis through a dual-stage pH-controlled fermentation process, thereby further increasing DA production. Compared with traditional constant-pH fermentation processes, dual-stage pH control not only adapts to the cell’s needs at different growth stages but also maintains optimal metabolic conditions at each stage, maximizing the synthesis of the target product. We also found that a mixed feeding strategy of Fe²^+^ and ascorbic acid significantly promoted DA synthesis, particularly when Fe²^+^ was at 40 mg/L and ascorbic acid at 20 mmol/L, resulting in a DA concentration of 22.58 g/L. However, excessive Fe²^+^ or ascorbic acid concentrations can be toxic to the cells, limiting DA synthesis. This highlights the importance of precisely controlling Fe²^+^ and ascorbic acid concentrations to maximize DA production.

Overall, this study successfully enhanced dopamine production levels through a multifaceted optimization strategy and provided a feasible solution for industrial-scale production. Compared with previous studies, our work optimized the gene expression system and improved DA synthesis efficiency by regulating cofactors and fermentation conditions. Future research should continue to explore global promoter optimization, more efficient cofactor supply systems, and fermentation process control to further enhance dopamine yield and production stability, supporting the application of DA in the pharmaceutical and biomedical fields.

## MATERIALS AND METHODS

### Strains, plasmids, and culture conditions

The plasmids and engineered strains used in this study are listed in Tables 2 and 3. *E. coli* DH5α was utilized for plasmid vector construction and cloning, and *E. coli* W3110 was used as the starting strain for metabolic engineering. As required by the experiment, ampicillin (50 µg/mL) and spectinomycin (50 µg/mL) were used during gene editing. The cultivation temperature of the engineered strains was adjusted according to experimental requirements.

**TABLE 2:** Strains used in this study

Strain	Description	Reference
*E. coli w3110*	Expression host	Lab stock
*E. coli* DH5α	Host for cloning	Lab stock
DA-1	*E. coli w3110, lacI*::xylA-*T7 RNA Polymerase*, △*tynA*	This study
DA-1–1	DA-1–1, *ylbE*::lac-*hpaBC*	This study
DA-2	DA-1–1, *yjiK*::lac-*HaDdc*	This study
DA-3	DA-1–1, *yjiK*::lac-*SsDdc*	This study
DA-4	DA-1–1,*yjiK*::lac-*HsDdc*	This study
DA-5	DA-1–1, *yjiK*::lac-*CfDdc*	This study
DA-6	DA-1–1, *yjiK*::lac-*DmDdc*	This study
DA-7	DA-1, *ylbE*:M1-93- *hpaBC*, *yjiK*::M1-93- *DmDdc*	This study
DA-8	DA-1, *ylbE*::M1-93- *hpaBC*, *yjiK*::trc- *DmDdc*	This study
DA-9	DA-1, *ylbE*::M1-93- *hpaBC*, *yjiK*::T7- *DmDdc*	This study
DA-10	DA-1, *ylbE*::trc- *hpaBC*, *yjiK*::M1-93- *DmDdc*	This study
DA-11	DA-1, *ylbE*::trc- *hpaBC*, *yjiK*::trc- *DmDdc*	This study
DA-12	DA-1, *ylbE*::trc- *hpaBC*, *yjiK*::T7- *DmDdc*	This study
DA-13	DA-1, *ylbE*::T7- *hpaBC*, *yjiK*::M1-93- *DmDdc*	This study
DA-14	DA-1, *ylbE*::T7- *hpaBC*, *yjiK*::trc- *DmDdc*	This study
DA-15	DA-1, *ylbE*::T7- *hpaBC*, *yjiK*::T7- *DmDdc*	This study
DA-16	DA-12, △*tyrR*, *ygaY*::trc-*aroG^fbr^*, *ycgH*::trc-*tyrA^fbr^*	This study
DA-17	DA-16, *yciQ*::trc-*aroE*, *yeeL*::trc-*tyrB*	This study
DA-18	DA-17, △*pykA*	This study
DA-19	DA-17, △*pykF*	This study
DA-20	DA-17, △*pykA*, △*pykF*	This study
DA-21	DA-18, *yncK*::trc- *pps*	This study
DA-22	DA-19, *yncK*::trc- *pps*	This study
DA-23	DA-22, *yeeP*::trc-*tktA*, *yjgX*::trc-*talB*	This study
DA-24	DA-23, *yghE*::trc-hpaBC, *mbhA*::T7-*DmDdc*	This study
DA-25	DA-24, *gapC*::trc-hpaBC, *rph*::T7-*DmDdc*	This study
DA-26	DA-25, *ilvG*::trc-*hpaBC*, *ycdN*::T7-*DmDdc*	This study
DA-27	DA-26, *yjiV*::trc-*fre*,	This study
DA-28	DA-27, △*adhE*	This study
DA-29	DA-28, *yncI*::trc-*gapA*	This study

**TABLE 3:** Plasmids used in this study

Plasmid	Description	Reference
pGRB	gRNA expression vector	Lab stock
pRed-cas9	Cas9 expression vector	Lab stock

### Acquisition of the target gene

Unless otherwise specified, all genes used in this study were derived from *E. coli* W3110. After codon optimization, GENEWIZ (Tianjin, China) artificially synthesized all heterologous target genes. GENEWIZ Biotech (Suzhou, China) synthesized all primers described in the supplementary material. For example, using *E. coli* W3110 genomic DNA as a template, the target gene was amplified using a U/D primer pair and heat-stable DNA polymerase.

### Genome editing

The CRISPR/Cas9 system was used for gene knockout and overexpression (48). As an example of the knockout of the *tyrR* gene. A 20 bp spacer sequence was obtained using CRISPR RGEN Tools (http://www.rgenome.net/). Target gRNA sequences were obtained from the Cas-Designer page of the CRISPR RGEN Tool website (http://www.rgenome.net/cas-designer/). Next, the flanking sequences required for recombination were added to both ends of the gRNA sequence, and a pair of complementary primers (tyrR-pGRB-U and tyrR-pGRB-D) was synthesized to form two reverse complementary ssDNA fragments. The two ssDNA fragments were annealed to form a dsDNA fragment containing the target gRNA sequence for plasmid recombination. The recombinant fragment was ligated into the linearized pGRB plasmid using a rapid cloning technique to construct the pGRB-tyrR plasmid. Primers tyrR-U-S/tyrR-U-A and tyrR-D-S/tyrR-D-A were designed (tyrR-U-A and tyrR-D-S contain partially homologous sequences) to amplify the upstream and downstream homologous fragments of tyrR, and the target DNA fragment (DNA-tyrR) was obtained by overlapping PCR. The pGRB-tyrR plasmid containing the targeted tyrR sequence and the DNA-tyrR fragment was then co-transformed into *E. coli* W3110 competent cells. Positive transformants were selected by colony PCR on an LB solid medium containing ampicillin and spectinomycin. The donor DNA was linked to the integrated gene through the upstream and downstream homologous arms when the target gene was incorporated into the genome. The remaining steps followed the same procedure as described above.

### Shake flask fermentation culture

The experimental strain was inoculated into a 500 mL shake flask containing 30 mL of seed medium and cultured at 36°C and 220 rpm until the biomass reached 14.0–16.0 (OD600 nm). Then, 3 mL of the seed culture was transferred into a 500 mL shake flask containing 30 mL fermentation medium and cultured at 36°C and 220 rpm for 24 h. During the fermentation process, ammonia solution (25%, vol/vol) was added based on the color changes of the phenol red indicator to maintain the pH at 7.0 ± 0.1. When glucose in the medium was depleted, a 60% (wt/vol) glucose solution was added to sustain fermentation. A sterile glucose solution (60%, wt/vol) was supplied once the glucose in the initial culture was depleted to meet the carbon source demands for cell growth and product synthesis. Xylose was added at a final concentration of 5 g/L at the start of fermentation to induce gene expression driven by the T7 promoter. The composition of the seed medium included 30 g/L glucose, 6.0 g/L yeast extract, 2.0 g/L peptone, 2 g/L citric acid, 3.0 g/L (NH4)_2_ SO_4_, 0.4 g/L MgSO_4_·7H_2_O, 0.5 g/L glutamic acid, 5.0 mg/L MnSO_4_, 0.5 mg/L VB_(1.3.5.12)_, and 2% (wt/vol) phenol red. The composition of the fermentation medium included 20 g/L glucose, 6.0 g/L yeast extract, 2.0 g/L peptone, 2 g/L citric acid, 3.0 g/L (NH4)_2_ SO_4_, 0.4 g/L MgSO_4_·7H_2_O, 0.5 g/L glutamic acid, 2 g/L KH_2_PO_4_, 5.0 mg/L MnSO_4_, 0.5 mg/L VB_(1.3.5.12)_, PLP 20 mg/mL, and 2% (wt/vol) phenol red.

### Batch-fed fermentation in a 5L bioreactor

The recombinant *E. coli* strain was fermented in a 5 L bioreactor (Biotech-5 BG; Baoxing Biotech Equipment Co., Ltd., China) with an initial volume of 2.0 L of seed medium. During the seed phase, the temperature, pH, and dissolved oxygen (DO) levels were maintained at 36°C, 7.0% ± 0.1%, and 30% ± 5%, respectively. The medium used in the 5 L bioreactor did not contain phenol red. When the OD600 nm reached 15% ± 1%, 20% of the seed culture was inoculated into the fermentation tank containing 1.6 L of fermentation medium. During the fed-batch fermentation, the temperature was maintained at 36°C, and dissolved oxygen (DO) levels were controlled by adjusting the stirring speed and airflow rate.

### Analytical methods

Optical density was measured at 600 nm using a UV spectrophotometer (UV 1800; Anxin Technology Instruments Co., Ltd., Shanghai, China) to monitor cell growth. The glucose concentration in the fermentation broth was measured using a 125 SBA-40 C biosensor analyzer (Shandong Academy of Sciences, China). NADH and NAD+ concentrations were measured using NADH/NAD+ analysis kits and WST-8 (S0175; Beyotime). The concentrations of dopamine and L-DOPA were determined by high-performance liquid chromatography (HPLC) (Prominence LC-20A, Shimadzu, Japan) equipped with an Innoval C18 column (4.6 mm × 250 mm, 5 µm). The detection temperature was set to 25°C, with the mobile phase consisting of a 12:88 (vol/vol) mixture of methanol and 10 mM potassium phosphate buffer (pH 6.5) and a flow rate of 1 mL/min.

## Data Availability

All experimental data and supporting materials in this study are publicly available and can be accessed through supplemental material or databases. Specific data can be requested by contacting the authors or retrieved from online public databases such as GenBank and KEGG. The accession numbers for the involved nucleotide and amino acid sequences are provided in the supplemental material. For any additional data requests, please contact the corresponding authors.

## SUPPLEMENTAL MATERIAL

Supplemental tables aem.00159-25-s0001.docx Tables S1 to S4.

ASM does not own the copyrights to Supplemental Material that may be linked to, or accessed through, an article. The authors have granted ASM a non-exclusive, world-wide license to publish the Supplemental Material files. Please contact the corresponding author directly for reuse.
