# Supplementary material for: Metabolic engineering of Escherichia coli for high-yield dopamine production via optimized fermentation strategies
Source: Appl Environ Microbiol. 2025 May 8;91(6):e00159-25. doi: 10.1128/aem.00159-25 (PMC12175537; doi:10.1128/aem.00159-25)
Supplement: Supplemental tables — Tables S1 to S4. [file aem.00159-25-s0001.docx]

**Supporting Information**

**Metabolic Engineering of *Escherichia coli* for High-Yield Dopamine Production via Optimized Fermentation Strategies**

**Xu Li,^a,b^ Yanghao Liu,^a,b^ Ling Ma,^a,b^ Wenjing Jiang^a,b^ Tangen Shi^a,b^ Lanxiao Li^a,b^  Changgeng Li^a,b^ Zhichao Chen^a,b^ Xiaoguang Fan^a,b*^ Qingyang Xu^a,b*^**

**^a^** College of Biotechnology, Tianjin University of Science & Technology, Tianjin, 300457, P. R. China.

**^b^** Key Laboratory of Industrial Fermentation Microbiology of the Ministry of Education, Tianjin University of Science & Technology, Tianjin, 300457, P. R. China

**^*^**Email: xiaoguangfan@tust.edu.cn, xuqingyang@tust.edu.cn.

**Supplementary Table S1**: The primers used in this study

| Primers | Sequences |
| --- | --- |
| lacI-pGRB-U | AGTCCTAGGTATAATACTAGTCGTCGAAGCCTGTAAAGCGGGTTTTAGAGCTAGAA |
| lacI-pGRB-D | TTCTAGCTCTAAAACCCGCTTTACAGGCTTCGACGACTAGTATTATACCTAGGACT |
| lacI-U-S | AGGCAGCGGAAAAGCATCT |
| lacI-U-A | CCCAGTTTCATCATTCCATTTTATTTTGCGAGCGAGCGCACACTTGTGAATTATCTCGGCAACGCCAATCAGCAAC |
| lacI-D-S | CGAAGGAAAGACCTGATGCTTTTCGTGCGCGCATAAAATACCTTGATACTGTGCCGGCGACGATACCGAAGACAGC |
| lacI-D-A | AGGGTTTTCCCAGTCACGA |
| xylA-U | GTTGCTGATTGGCGTTGCCGAGATAATTCACAAGTGTGCGCTCGCTCGCAAAATAAAATGGAATGATGAAACTGGG |
| xylA-D | GCCTCTTCCAGTTAGTAAATCCCATGGTGTAGGGCCTTCTGTAGTTAGAGGACAGTTTTAATAAGTAACAATCACCGCGA |
| T7RNP-U | TCGCGGTGATTGTTACTTATTAAAACTGTCCTCTAACTACAGAAGGCCCTACACCATGGGATTTACTAACTGGAAGAGGC |
| T7RNP-D | GCGCACGAAAAGCATCAGGTCTTTCCTTCGAAGGGGATCCGGAGTCGTATTGATTTGGCGTTACGCGAACGCGAAGTCCG |
| tynA-pGRB-U | AGTCCTAGGTATAATACTAGTTTACTGGCGATATGATTCACGTTTTAGAGCTAGAA |
| tynA-pGRB-D | TTCTAGCTCTAAAACGTGAATCATATCGCCAGTAAACTAGTATTATACCTAGGACT |
| tynA-U-S | CGTGTCCACTATTGCTGGGTAA |
| tynA-U-A | CCCATTCGGTCGGCATAATGGCGAAATCATCCAGCAACA |
| tynA-D-S | TGTTGCTGGATGATTTCGCCATTATGCCGACCGAATGGG |
| tynA-D-A | CCGGAGAGGGGTATTATGTTG |
| ylbE-pGRB-U | AGTCCTAGGTATAATACTAGTACACTGGCTGGATGTGCAACGTTTTAGAGCTAGAA |
| ylbE-pGRB-D | TTCTAGCTCTAAAACGTTGCACATCCAGCCAGTGTACTAGTATTATACCTAGGACT |
| yjiK-pGRB-U | AGTCCTAGGTATAATACTAGTTACGGGGTTTCTCCGCGTTAGTTTTAGAGCTAGAA |
| yjiK-pGRB-D | TTCTAGCTCTAAAACTAACGCGGAGAAACCCCGTAACTAGTATTATACCTAGGACT |
| ylbE-U-S | ACCCAACCTTACGCAACCAG |
| yblE-U-A(lac) | GAAATTGTTATCCGCTCACAATTCCACACAACATACGAGCCGGAAGCATAAAGTGTAAATTGTTCGATAACCGCAGCATTG |
| yblE-D-S | CTGGGCCTTTCGTTTTATCTGTTGTTTGTCGGTGAACGCTCTCCTGAGTAGGACAAATCGCTGGCGTGCTTTGAACAGGC |
| yblE-D-A | GGCGTAACTCAGCAGGCAG |
| hpaBC-lac-UP | TTGTGAGCGGATAACAATTTCACACAGGAAACAGCTATGAAACCAGAAGATTTCCGCGCCAGTACCCAACG |
| hpaBC-lac-DN | CAGATAAAACGAAAGGCCCAGTCTTTCGACTGAGCCTTTCGTTTTATTTGTTAAATCGCAGCTTCCATTTCC |
| yjiK-U-S | GAATCTCACCTGCGAATGCC |
| yjiK-U-A(lac) | TGTGTGAAATTGTTATCCGCTCACAATTCCACACAACATACGAGCCGGAAGCATAAAGTGTAAAACACCATCCTGCTGGC |
| yjiK-D-S | AAAGACTGGGCCTTTCGTTTTATCTGTTGTTTGTCGGTGAACGCTCTCCTGAGTAGGACAAATTTGCGATGGATGCCTCTGGT |
| yjiK-D-A | CCTAAGGTCAGAACCAGCATC |
| HaDdc-lac-UP | CCGGCTCGTATGTTGTGTGGAATTGTGAGCGGATAACAATTTCACACAGGAAACAGCTAAAACCCAGTGAAGGTAACC |
| HaDdc-lac-DN | CACCGACAAACAACAGATAAAACGAAAGGCCCAGTCTTTCGACTGAGCCTTTCGTTTTATTTGAGAAATCATGAACTTGTC |
| SsDdc-lac-UP | CCGGCTCGTATGTTGTGTGGAATTGTGAGCGGATAACAATTTCACACAGGAAACAGCTATGAACGCCAGTGATTTTCG |
| SsDdc-lac-DN | CACCGACAAACAACAGATAAAACGAAAGGCCCAGTCTTTCGACTGAGCCTTTCGTTTTATTTGTTAACTCTTAATTTCTGC |
| HsDdc-lac-UP | CCGGCTCGTATGTTGTGTGGAATTGTGAGCGGATAACAATTTCACACAGGAAACAGCTATGAACGCCAGTGAATTTCG |
| HsDdc-lac-DN | CCGACAAACAACAGATAAAACGAAAGGCCCAGTCTTTCGACTGAGCCTTTCGTTTTATTTGTTATTCGCGTTCCGCGCGCA |
| CfDdc-lac-UP | CCGGCTCGTATGTTGTGTGGAATTGTGAGCGGATAACAATTTCACACAGGAAACAGCTTAGGGGTATATGGAGAACATTC |
| CfDdc-lac-DN | CACCGACAAACAACAGATAAAACGAAAGGCCCAGTCTTTCGACTGAGCCTTTCGTTTTATTTGTTTTTTTTTTTTTTTTTG |
| DmDdc-lac-UP | CCGGCTCGTATGTTGTGTGGAATTGTGAGCGGATAACAATTTCACACAGGAAACAGCTATGAGCCATATTCCGATTAG |
| Dmddc-lac-DN | CCGACAAACAACAGATAAAACGAAAGGCCCAGTCTTTCGACTGAGCCTTTCGTTTTATTTGTTACTGTTCTTGTTCCATTTC |
| ylbE-U-A(M1-93) | GCTAACAATACGGGCTCAATTATATCAACGTTGTTATCTCTTGTCAACACCGCCAGAGATAATTGTTCGATAACCGCAGCATTG |
| hpaBC-M1-93-UP | GAGCCCGTATTGTTAGCATGTACGTTTAAACCAGGAAACAGCTATGAAACCAGAAGATTTCCGCGCCAGTACCCAACG |
| yjiK-U-A(M1-93) | ATGCTAACAATACGGGCTCAATTATATCAACGTTGTTATCTCTTGTCAACACCGCCAGAGATAAACACCATCCTGCTGGCAATAAA |
| DmDdc-M1-93-UP | CAACGTTGATATAATTGAGCCCGTATTGTTAGCATGTACGTTTAAACCAGGAAACAGCTATGAGCCATATTCCGATTAGC |
| yjiK-U-A(trc) | TGTGTGAAATTGTTATCCGCTCACAATTCCACACATTATACGAGCCGGATGATTAATTGTCAAACACCATCCTGCTGGCAATAAA |
| DmDdc-trc-UP | CGTATAATGTGTGGAATTGTGAGCGGATAACAATTTCACACAGGAAACAGACCATGAGCCATATTCCGATTAGCAACA |
| yjiK-U-A(T7) | CAAAATTATTTCTAGACCCTATAGTGAGTCGTATTAACACCATCCTGCTGGCAATAAATTGACTC |
| DmDdc-T7-UP | CTCACTATAGGGTCTAGAAATAATTTTGTTTAACTTTAAGAAGGAGATATACCATGAGCCATATTCCGATTAGCAACA |
| yblE-U-A(trc) | GAAATTGTTATCCGCTCACAATTCCACACATTATACGAGCCGGATGATTAATTGTCAATTGTTCGATAACCGCAGCATTG |
| hpaBC-trc-UP | TTGTGAGCGGATAACAATTTCACACAGGAAACAGACCATGAAACCAGAAGATTTCCGCGCCAGTACCCAACG |
| yblE-U-A(T7) | ATATCTCCTTCTTAAAGTTAAACAAAATTATTTCTAGACCCTATAGTGAGTCGTATTATTGTTCGATAACCGCAGCATTG |
| hpaBC-T7-UP | GGGTCTAGAAATAATTTTGTTTAACTTTAAGAAGGAGATATACCATGAAACCAGAAGATTTCCGCGCCAGTACCCAACG |
| tyrR-pGRB-U | AGTCCTAGGTATAATACTAGTACACGTCCTGACCGGTGCGGGTTTTAGAGCTAGAA |
| tyrR-pGRB-D | TTCTAGCTCTAAAACCCGCACCGGTCAGGACGTGTACTAGTATTATACCTAGGACT |
| tyrR-U-S | ATCTTTACGCCGAAGTGCC |
| tyrR-U-A | ACCGTCCAGTTGTGTCAGTCTCAACGCCAGATGCTCAC |
| tyrR-D-S | GTGAGCATCTGGCGTTGAGACTGACACAACTGGACGGT |
| tyrR-D-A | CCTCTCCACTTTCCGTAAC |
| ygaY-pGRB-U | AGTCCTAGGTATAATACTAGTCACTGATGGCGCTGGCATTAGTTTTAGAGCTAGAA |
| ygaY-pGRB-D | TTCTAGCTCTAAAACTAATGCCAGCGCCATCAGTGACTAGTATTATACCTAGGACT |
| ygaY-U-S | CCTACAAACCACATCGCACATT |
| ygaY-U-A | AATTGTTATCCGCTCACAATTCCACACATTATACGAGCCGGATGATTAATTGTCAAACACCGAAGCAACCCAAAAG |
| ygaY-D-S | AAAGACTGGGCCTTTCGTTTTATCTGTTGTTTGTCGGTGAACGCTCTCCTGAGTAGGACAAATTTGCTTGCCGCTCCACC |
| ygaY-D-A | GGAGTAGGGCTTTCCATAGAGTGT |
| aroG^fbr^-trc-UP | GTGTGGAATTGTGAGCGGATAACAATTTCACACAGGAAACAGACCATGAATTATCAGAACGACGATTTACGCATCAAAGA |
| aroG^fbr^-trc-DN | AGATAAAACGAAAGGCCCAGTCTTTCGACTGAGCCTTTCGTTTTATTTGTTACCCGCGACGCGCTTTTACTGCATTCGCC |
| ycgH-pGRB-U | AGTCCTAGGTATAATACTAGTTATGCGTCTGAACGACCGTGGTTTTAGAGCTAGAA |
| ycgH-pGRB-D | TTCTAGCTCTAAAACCACGGTCGTTCAGACGCATAACTAGTATTATACCTAGGACT |
| ycgH-U-S | TAAACTCGTCAGCGGCACAA |
| ycgH-U-A | AATTGTTATCCGCTCACAATTCCACACATTATACGAGCCGGATGATTAATTGTCAAGGTAGGCGTTTCTGTTGATTCTG |
| ycgH-D-S | AAAGACTGGGCCTTTCGTTTTATCTGTTGTTTGTCGGTGAACGCTCTCCTGAGTAGGACAAATGCGTGTCGGATTATCGTTCG |
| ycgH-D-A | GATTCAGGTTGCCATTTACGC |
| tyrA^fbr^-trc-UP | GTGGAATTGTGAGCGGATAACAATTTCACACAGGAAACAGACCATGGTTGCTGAATTGACCGCATTACGCGATC |
| tyrA^fbr^-trc-DN | CAGATAAAACGAAAGGCCCAGTCTTTCGACTGAGCCTTTCGTTTTATTTGTTACTGGCGATTGTCATTCGCC |
| yciQ-pGRB-U | AGTCCTAGGTATAATACTAGTCTTCACGATAAAACGGATCGGTTTTAGAGCTAGAA |
| yciQ--pGRB-D | TTCTAGCTCTAAAACCGATCCGTTTTATCGTGAAGACTAGTATTATACCTAGGACT |
| yciQ-U-S | TTACTTGAAGCATTGGGCGAAC |
| yciQ-U-A | AATTGTTATCCGCTCACAATTCCACACATTATACGAGCCGGATGATTAATTGTCAACCAGTCAAGATGCCAGGGTTC |
| yciQ-D-S | AAAGACTGGGCCTTTCGTTTTATCTGTTGTTTGTCGGTGAACGCTCTCCTGAGTAGGACAAATGTCTGACAAGAACCAGCAAATCCT |
| yciQ-D-A | ATAGCTTCACCGTGGGCATAAC |
| aroE-trc-UP | GTATAATGTGTGGAATTGTGAGCGGATAACAATTTCACACAGGAAACAGACCATGGAAACCTATGCTGTTTTTGGTAATC |
| aroE--trc-DN | AACAGATAAAACGAAAGGCCCAGTCTTTCGACTGAGCCTTTCGTTTTATTTGTCACGCGGACAATTCCTCCTGCAATTGC |
| yeeL-pGRB-U | AGTCCTAGGTATAATACTAGTAACACAGCAATACGGTACGCGTTTTAGAGCTAGAA |
| yeeL-pGRB-D | TTCTAGCTCTAAAACGCGTACCGTATTGCTGTGTTACTAGTATTATACCTAGGACT |
| yeeL-U-S | TTCATCGGGACGAGTGGAGA |
| yeeL-U-A | TCCACACATTATACGAGCCGGATGATTAATTGTCAACCATAGCATCGCCAATCTGATCGGG |
| yeeL-D-S | AAAGACTGGGCCTTTCGTTTTATCTGTTGTTTGTCGGTGAACGCTCTCCTGAGTAGGACAAATACCCAAAGGTGAAGATA |
| yeeL-D-A | CATTCCCTCTACAGAACTAG |
| tyrB--trc-UP | CCGGCTCGTATAATGTGTGGAATTGTGAGCGGATAACAATTTCACACAGGAAACAGACCGTGTTTCAAAAAGTTGACGCC |
| tyrB-trc-DN | TATCTTCACCTTTGGGTATTTGTCCTACTCAGGAGAGCGTTCACCGACAAACAACAGATAAAACGAAAGGCCCAGTCTTT |
| pykA-pGRB-U | AGTCCTAGGTATAATACTAGTCAAACTTGGCGGCGGTTTGTGTTTTAGAGCTAGAA |
| pykA-pGRB-D | TTCTAGCTCTAAAACACAAACCGCCGCCAAGTTTGACTAGTATTATACCTAGGACT |
| pykA-U-S | CGACTGTCACTGTCCTAAT |
| pykA-U-A | GAGAGCAGTCAGGTTCAGCGGATACACGGATTTTGGGC |
| pykA-D-S | GCCCAAAATCCGTGTATCCGCTGAACCTGACTGCTCTC |
| pykA-D-A | TTTATGATGGCAAGACGCA |
| pykF-pGRB-U | AGTCCTAGGTATAATACTAGTGACAAACAGGACCTGATCTTGTTTTAGAGCTAGAA |
| pykF-pGRB-D | TTCTAGCTCTAAAACAAGATCAGGTCCTGTTTGTCACTAGTATTATACCTAGGACT |
| pykF-U-S | ATCCTTAGAGCGAGGCACC |
| pykF-U-A | CCAGTTCTTTACCCAGACGCAGGATAGCGGCGGTTTTA |
| pykF-D-S | TAAAACCGCCGCTATCCTGCGTCTGGGTAAAGAACTGG |
| pykF-D-A | GATCGTTCGCTCAAAGAAGC |
| yncK-pGRB-U | AGTCCTAGGTATAATACTAGTATCGACCAGATATTGCATTCGTTTTAGAGCTAGAA |
| yncK-pGRB-D | TTCTAGCTCTAAAACGAATGCAATATCTGGTCGATACTAGTATTATACCTAGGACT |
| yncK-D-S | AAAGACTGGGCCTTTCGTTTTATCTGTTGTTTGTCGGTGAACGCTCTCCTGAGTAGGACAAATGCCCTGAAGGATGGGGTTTTAC |
| yncK-D-A | CGCAGCGTAAACCACTGGTTAC |
| pps-trc-UP | GGAATTGTGAGCGGATAACAATTTCACACAGGAAACAGACCATGTCCAACAATGGCTCGTCACCGCTGGTGC |
| pps-trc-DN | CAGATAAAACGAAAGGCCCAGTCTTTCGACTGAGCCTTTCGTTTTATTTGTTATTTCTTCAGTTCAGCCAGGCTTAACC |
| yeeP-pGRB-U | AGTCCTAGGTATAATACTAGTCGTATCGGCCCGCACCGGCTGTTTTAGAGCTAGAA |
| yeeP-pGRB-D | TTCTAGCTCTAAAACAGCCGGTGCGGGCCGATACGACTAGTATTATACCTAGGACT |
| yeeP-U-S | GGTCAGGAGGTAACTTATCAGCG |
| yeeP-U-A | AATTGTTATCCGCTCACAATTCCACACATTATACGAGCCGGATGATTAATTGTCAAATGGCAGGGCTCCGTTTT |
| yeeP-D-S | AAAGACTGGGCCTTTCGTTTTATCTGTTGTTTGTCGGTGAACGCTCTCCTGAGTAGGACAAATGAACTGGATTTTCTTCTGAACCTGT |
| yeeP-D-A | ACGATGTCAGCAGCCAGCA |
| tktA-trc-UP | GGAATTGTGAGCGGATAACAATTTCACACAGGAAACAGACCATGTCCTCACGTAAAGAGCTTGCC |
| tktA-trc-DN | GATAAAACGAAAGGCCCAGTCTTTCGACTGAGCCTTTCGTTTTATTTGTTACAGCAGTTCTTTTGCTTTCGC |
| yjgX-pGRB-U | AGTCCTAGGTATAATACTAGTTCGCGACCACCGTAACTGGCGTTTTAGAGCTAGAA |
| yjgX-pGRB-D | TTCTAGCTCTAAAACGCCAGTTACGGTGGTCGCGAACTAGTATTATACCTAGGACT |
| yjgX-U-S | GGAAGTCAACGGGTTATGCG |
| yjgX-U-A | AATTGTTATCCGCTCACAATTCCACACATTATACGAGCCGGATGATTAATTGTCAAAAAATCACCACGAATACCAGAATC |
| yjgX-D-S | AAAGACTGGGCCTTTCGTTTTATCTGTTGTTTGTCGGTGAACGCTCTCCTGAGTAGGACAAATACAGTGTCTTCCCTGAGCCG |
| yjgX-D-A | GGCGAAGGATACCATCAAGC |
| talB-trc-UP | CGTATAATGTGTGGAATTGTGAGCGGATAACAATTTCACACAGGAAACAGACCATGACGGACAAATTGACCTCCCTTC |
| talB-trc-DN | CCGACAAACAACAGATAAAACGAAAGGCCCAGTCTTTCGACTGAGCCTTTCGTTTTATTTGTTACAGCAGATCGCCGATCATT |
| yghE-pGRB-U | AGTCCTAGGTATAATACTAGTGCTGAAAAAATATCGCCCACGTTTTAGAGCTAGAA |
| yghE-pGRB-D | TTCTAGCTCTAAAACGTGGGCGATATTTTTTCAGCACTAGTATTATACCTAGGACT |
| yghE-U-S | GTCAGGCACTGGCGAAAGAT |
| yghE-U-A | AATTGTTATCCGCTCACAATTCCACACATTATACGAGCCGGATGATTAATTGTCAACGCAAGCCATAAACCCACA |
| yghE-D-S | CTGGGCCTTTCGTTTTATCTGTTGTTTGTCGGTGAACGCTCTCCTGAGTAGGACAAATTTCCGACATCGAAATGCGT |
| yghE-D-A | AGGCGTTGTTGTGGCAGATT |
| mbhA-pGRB-U | AGTCCTAGGTATAATACTAGTGCGTGATGTGAATGAGAAAAGTTTTAGAGCTAGAA |
| mbhA-pGRB-D | TTCTAGCTCTAAAACTTTTCTCATTCACATCACGCACTAGTATTATACCTAGGACT |
| mbhA-U-S | GCCAGCACGAACATAATCCC |
| mbhA-U-A | AATTGTTATCCGCTCACAATTCCACACATTATACGAGCCGGATGATTAATTGTCAACACGGTGGCAGGTTTTGG |
| mbhA-D-S | AAAGACTGGGCCTTTCGTTTTATCTGTTGTTTGTCGGTGAACGCTCTCCTGAGTAGGACAAATGACCAAAAGTGCGTCCGATAC |
| mbhA-D-A | CGGCGTAATCACAAACTGGC |
| gapC-pGRB-U | AGTCCTAGGTATAATACTAGTAAAGCCTTGCATGACAGTTTCGGGTTTTAGAGCTAGAA |
| gapC-pGRB-D | TTCTAGCTCTAAAACCCGAAACTGTCATGCAAGGCTTTACTAGTATTATACCTAGGACT |
| gapC-U-S | TGGGAAGAAACCACGAAACT |
| gapC-U-A | GAAATTGTTATCCGCTCACAATTCCACACATTATACGAGCCGGATGATTAATTGTCAATGTTTCAGCAGGTAGGCGAG |
| gapC-D-S | GGGCCTTTCGTTTTATCTGTTGTTTGTCGGTGAACGCTCTCCTGAGTAGGACAAATAAAACGGTCGCCTGGTACG |
| gapC-D-A | TTATCCGCCGACATTGCTGC |
| rph-pGRB-U | AGTCCTAGGTATAATACTAGTGGCTGGATCACCGCAGAGTAGTTTTAGAGCTAGAA |
| rph-pGRB-D | TTCTAGCTCTAAAACTACTCTGCGGTGATCCAGCCACTAGTATTATACCTAGGACT |
| rph-U-S | ATAGCGCAGGGTACATTCCACT |
| rph-U-A | AATTGTTATCCGCTCACAATTCCACACATTATACGAGCCGGATGATTAATTGTCAACCTTCTTCAATAGAGGCGGTACA |
| rph-D-S | AAAGACTGGGCCTTTCGTTTTATCTGTTGTTTGTCGGTGAACGCTCTCCTGAGTAGGACAAATTGCCGCAGAGACCGACAT |
| rph-D-A | ACAGCGGTTGTGGTGGCA |
| ilvG-pGRB-U | AGTCCTAGGTATAATACTAGTTATCGGCACTGACGCATTTCGTTTTAGAGCTAGAA |
| ilvG-pGRB-D | TTCTAGCTCTAAAACGAAATGCGTCAGTGCCGATAACTAGTATTATACCTAGGACT |
| ilvG-U-S | ACCGAGGAGCAGACAATGAATAA |
| ilvG-U-A | AATTGTTATCCGCTCACAATTCCACACATTATACGAGCCGGATGATTAATTGTCAAGGTGATGGCAACAACAGGGA |
| ilvG-D-S | AAAGACTGGGCCTTTCGTTTTATCTGTTGTTTGTCGGTGAACGCTCTCCTGAGTAGGACAAATCTATCTACGCGCCGTTGTTGT |
| ilvG-D-A | GCGCTGGCTAACATGAGGAA |
| ycdN-pGRB-U | AGTCCTAGGTATAATACTAGTGCGTGGAAATCATCATGGCTGTTTTAGAGCTAGAA |
| ycdN-pGRB-D | TTCTAGCTCTAAAACAGCCATGATGATTTCCACGCACTAGTATTATACCTAGGACT |
| ycdN-U-S | GATTTTGACGCCACCAACACC |
| ycdN-U-A | AATTGTTATCCGCTCACAATTCCACACATTATACGAGCCGGATGATTAATTGTCAACCAATCCACATCACACAATCCAT |
| ycdN-D-S | AAAGACTGGGCCTTTCGTTTTATCTGTTGTTTGTCGGTGAACGCTCTCCTGAGTAGGACAAATGAAGGGATTTTTGGCTATCAGGA |
| ycdN-D-A | CATATCGTATTCGCCAGGCTG |
| yjiV-pGRB-U | AGTCCTAGGTATAATACTAGTGGCTGGATCGATTTCGTTCCGTTTTAGAGCTAGAA |
| yjiV-pGRB-D | TTCTAGCTCTAAAACGGAACGAAATCGATCCAGCCACTAGTATTATACCTAGGACT |
| yjiV-U-S | TGTGACTGTGGAAGCCCTGTAT |
| YjiV-U-A | AATTGTTATCCGCTCACAATTCCACACATTATACGAGCCGGATGATTAATTGTCAAATTCGGGCTGTCCCTTGTC |
| YjiV-D-S | AAAGACTGGGCCTTTCGTTTTATCTGTTGTTTGTCGGTGAACGCTCTCCTGAGTAGGACAAATGTGGCACCTGAATGACGAACT |
| YjiV-D-A | TGGCGACATTCCCTTCCTT |
| adhE-pGRB-u | AGTCCTAGGTATAATACTAGTAGCAGAGAAACTGGTTGCTAGTTTTAGAGCTAGAA |
| adhE-pGRB-d | TTCTAGCTCTAAAACTAGCAACCAGTTTCTCTGCTACTAGTATTATACCTAGGACT |
| adhE-U-S | GAAGACGACACTTTTGGTACCATCA |
| adhE-U-A | TGGTTTGAAGGAGTTTGCCAGCTGTATGCGGCTTTAAC |
| adhE-D-S | GTTAAAGCCGCATACAGCTGGCAAACTCCTTCAAACCA |
| adhE-D-A | AGACCACCGAAAGCACACAGG |
| yncI-pGRB-u | CTGAACAACATCATATTTAATTACAGGACGCACTGTGGAGTGGCTAGCATAACCCCTTGGGGC |
| yncI-pGRB-d | GCCCCAAGGGGTTATGCTAGCCACTCCACAGTGCGTCCTGTAATTAAATATGATGTTGTTCAG |
| yncI-U-S | GGGCAACTCTTCGGGTTAG |
| yncI-U-A | CCGCTCACAATTCCACACATTATACGAGCCGGATGATTAATTGTCAATGTAGGCGTTAAAGCAAAGATGAA |
| yncI-D-S | GGGCCTTTCGTTTTATCTGTTGTTTGTCGGTGAACGCTCTCCTGAGTAGGACAAATTTGGGTGTTAGATGTAAAAATGAATG |
| yncI-D-A | GCAATGACGTCTTTATCATCTGAAG |
| gapA-trc-UP | CGTATAATGTGTGGAATTGTGAGCGGATAACAATTTCACACAGGAAACAGACCATGACTATCAAAGTAGGTATCAACG |
| gapA-trc-DN | CCGACAAACAACAGATAAAACGAAAGGCCCAGTCTTTCGACTGAGCCTTTCGTTTTATTTGTTATTTGGAGATGTGAGCG |

**Supplementary Table S2**: the promoters used in this study

| Promoter | Sequences |
| --- | --- |
| lac | TTTACACTTTATGCTTCCGGCTCGTATGTTGTGTGGAATTGTGAGCGGATAACAATTTCACACAGGAAACAGCT |
| M1-93 | TTATCTCTGGCGGTGTTGACAAGAGATAACAACGTTGATATAATTGAGCCCGTATTGTTAGCATGTACGTTTAAACCAGGAAACAGCT |
| trc | TTGACAATTAATCATCCGGCTCGTATAATGTGTGGAATTGTGAGCGGATAACAATTTCACACAGGAAACAGACC |
| T7 | TAATACGACTCACTATAGGGTCTAGAAATAATTTTGTTTAACTTTAAGAAGGAGATATACC |
| terminator | CAAATAAAACGAAAGGCTCAGTCGAAAGACTGGGCCTTTCGTTTTATCTGTTGTTTGTCGGTGAACGCTCTCCTGAGTAGGACAAAT |

**Supplementary Table S3**: The mutated gene sequences involved in this study

| Gene | Sequences |
| --- | --- |
| *aroG^fbr^* | ATGAATTATCAGAACGACGATTTACGCATCAAAGAAATCAAAGAGTTACTTCCTCCTGTCGCATTGCTGGAAAAATTCCCCGCTACTGAAAATGCCGCGAATACGGTTGCCCATGCCCGAAAAGCGATCCATAAGATCCTGAAAGGTAATGATGATCGCCTGTTGGTTGTGATTGGCCCATGCTCAATTCATGATCCTGTCGCGGCAAAAGAGTATGCCACTCGCTTGCTGGCGCTGCGTGAAGAGCTGAAAGATGAGCTGGAAATCGTAATGCGCGTCTATTTTGAAAAGCCGCGTACCACGGTGGGCTGGAAAGGGCTGATTAACGATCCGCATATGGATAATAGCTTCCAGATCAACGACGGTCTGCGTATAGCCCGTAAATTGCTGCTTGATATTAACGACAGCGGTCTGCCAGCGGCAGGTGAGTTTCTCAATATGATCACCCCACAATATCTCGCTGACCTGATGAGCTGGGGCGCAATTGGCGCACGTACCACCGAATCGCAGGTGCACCGCGAACTGGCATCAGGGCTTTCTTGTCCGGTCGGCTTCAAAAATGGCACCGACGGTACGATTAAAGTGGCTATCGATGCCATTAATGCCGCCGGTGCGCCGCACTGCTTCCTGTCCGTAACGAAATGGGGGCATTCGGCGATTGTGAATACCAGCGGTAACGGCGATTGCCATATCATTCTGCGCGGCGGTAAAGAGCCTAACTACAGCGCGAAGCACGTTGCTGAAGTGAAAGAAGGGCTGAACAAAGCAGGCCTGCCAGCACAGGTGATGATCGATTTCAGCCATGCTAACTCGTCCAAACAATTCAAAAAGCAGATGGATGTTTGTGCTGACGTTTGCCAGCAGATTGCCGGTGGCGAAAAGGCCATTATTGGCGTGATGGTGGAAAGCCATCTGGTGGAAGGCAATCAGAGCCTCGAGAGCGGGGAGCCGCTGGCCTACGGTAAGAGCATCACCGATGCCTGCATCGGCTGGGAAGATACCGATGCTCTGTTACGTCAACTGGCGAATGCAGTAAAAGCGCGTCGCGGGTAA |
| *tyrA^fbr^* | ATGGTTGCTGAATTGACCGCATTACGCGATCAAATTGATGAAGTCGATAAAGCGCTGCTGAATTTATTAGCGAAGCGTCTGGAACTGGTTGCTGAAGTGGGCGAGGTGAAAAGCCGCTTTGGACTGCCTATTTATGTTCCGGAGCGCGAGGCATCTATATTGGCCTCGCGTCGTGCAGAGGCGGAAGCTCTGGGTGTACCGCCAGATCTGATTGAGGATGTTTTGCGTCGGGTGATGCGTGAATCTTACTCCAGTGAAAACGACAAAGGATTTAAAACACTTTGTCCGTCACTGCGTCCGGTGGTTATCGTCGGCGGTGGCGGTCAGATGGGACGCCTGTTCGAGAAGATGCTGACCCTCTCGGGTTATCAGGTGCGGATTCTGGAGCAACATGACTGGGATCGAGCGGCTGATATTGTTGCCGATGCCGGAATGGTGATTGTTAGTGTGCCAATCCACGTTACTGAGCAAGTTATTGGCAAATTACCGCCTTTACCGAAAGATTGTATTCTGGTCGATCTGGCATCAGTGAAAAATGGGCCATTACAGGCCATGCTGGTGGCGCATGATGGTCCGGTGCTGGGGCTACACCCGATGTTCGGTCCGGACAGCGGTAGCCTGGCAAAGCAAGTTGTGGTCTGGTGTGATGGACGTAAACCGGAAGCATACCAATGGTTTCTGGAGCAAATTCAGGTCTGGGGCGCTCGGCTGCATCGTATTAGCGCCGTCGAGCACGATCAGAATATGGCGTTTATTCAGGCACTGCGCCACTTTGCTACTTTTGCTTACGGGCTGCACCTGGCAGAAGAAAATGTTCAGCTTGAGCAACTTCTGGCGCTCTCTTCGCCGATTTACCGCCTTGAGCTGGCGATGGTCGGGCGACTGTTTGCTCAGGATCCGCAGCTTTATGCCGACATCATTATGTCGTCAGAGCGTAATCTGGCGTTAATCAAACGTTACTATAAGCGTTTCGGCGAGGCGATTGAGTTGCTGGAGCAGGGCGATAAGCAGGCGTTTATTGACAGTTTCCGCAAGGTGGAGCACTGGTTCGGCGATTACGTACAGCGTTTTCAGAGTGAAAGCCGCGTGTTATTGCGTCAGGCGAATGACAATCGCCAGTAA |

**Supplementary Table S4**: The amino acid sequences of the decarboxylases involved in this study

| Gene | Sequences |
| --- | --- |
| HaDdc  ([AMQ13055.1](https://www.ncbi.nlm.nih.gov/protein/AMQ13055.1?report=genbank&log$=prottop&blast_rank=1&RID=W6PU3FCS013)) | MEANQFRDFGKAMIDYVANYLENIRERRVLPTVEPGYLRPLLPSEAPQKPDTWQEVMADIEKVIMPGVTHWHSPKFHAYFPTANSYPAIVADILSDGIACIGFSWIASPACTELEVVMMDWLGKMIGLPEEFLACSGGKGGGVIQGTASEATLVALLGAKARAIHHVKKEHPDWKDADIA  EKLVGYTSSQSHSSVERAGLLGGVKLRGLPTDESNRLRGDTLERAIKEDREAGLIPFYVVATLGTTSSCTFDNLEEIGPVCNVNKVWLHIDAAYAGAAFTCPEYRYLMKGVEMADSFDFNPHKWMLVTFDCSAMWLKDPNWLVDAFNVDPLYLKHDQQGSAPDYRHWQIQLGRRFRALKIWFVLRLYGVENIQKHIRKQIGLAHHFEDLVKSDDRFEVTEEVLMGLVCFRLKGQSNEVNERLLKRINARGTIHLVPSKIREMYFLRMAVCSRLTEKEDMDLSWKEVRESADDILGE |
| SsDdc  (NP_999019.2) | MNASDFRRRGKEMVDYMADYLEGIEGRQVYPDVQPGYLRPLIPATAPQEPDTFEDILQDVEKIIMPGVTHWHSPYFFAYFPTASSYPAMLADMLCGAIGCIGFSWAASPACTELETVMMDWLGKMLQLPEAFLAGEAGEGGGVIQGSASEATLVALLAARTKVTRRLQAASPGLTQGAVLEKLVAYASDQAHSSVERAGLIGGVKLKAIPSDGKFAMRASALQEALERDKAAGLIPFFVVATLGTTSCCSFDNLLEVGPICHEEDIWLHVDAAYAGSAFICPEFRHLLNGVEFADSFNFNPHKWLLVNFDCSAMWVKRRTDLTGAFKLDPVYLKHSHQGSGLITDYRHWQLPLGRRFRSLKMWFVFRMYGVKGLQAYIRKHVQLSHEFEAFVLQDPRFEVCAEVTLGLVCFRLKGSDGLNEALLERINSARKIHLVPCRLRGQFVLRFAICSRKVESGHVRLAWEHIRGLAAELLAAEEGKAEIKS |
| HsDdc  (NP_000781.2) | MNASEFRRRGKEMVDYMANYMEGIEGRQVYPDVEPGYLRPLIPAAAPQEPDTFEDIINDVEKIIMPGVTHWHSPYFFAYFPTASSYPAMLADMLCGAIGCIGFSWAASPACTELETVMMDWLGKMLELPKAFLNEKAGEGGGVIQGSASEATLVALLAARTKVIHRLQAASPELTQAAIMEKLVAYSSDQAHSSVERAGLIGGVKLKAIPSDGNFAMRASALQEALERDKAAGLIPFFMVATLGTTTCCSFDNLLEVGPICNKEDIWLHVDAAYAGSAFICPEFRHLLNGVEFADSFNFNPHKWLLVNFDCSAMWVKKRTDLTGAFRLDPTYLKHSHQDSGLITDYRHWQIPLGRRFRSLKMWFVFRMYGVKGLQAYIRKHVQLSHEFESLVRQDPRFEICVEVILGLVCFRLKGSNKVNEALLQRINSAKKIHLVPCHLRDKFVLRFAICSRTVESAHVQRAWEHIKELAADVLRAERE |
| CfDdc  (ADP08788) | MENIHNRRVIPEVEPGYLKQLLPDTAPEMSECFDDIMKDVERTIMPGITHWQHPHFHAYFPSGNSYPSILGDMLSDAIGCIGFSWASSPACTELEAITMDWLGKMMGLPNIFLHGSGEGGGVIQGSASECILVTLLAARHHALQERESMSPLIRNDSNLPKLVAYCSKLSHSCVEKAGMLGFVHLRQLDVDDNLSLRGNVLEAATQEDKKLGFIPFYVCATLGTTACCSFDNIAELGEVCVRENIWLHVDAAYAGNALICPEFQHLIKGAENLTSFSCNPNKWMLVNFDCSLLWVRDRLMLTSSMTVDPLYLQHKHEDQTIDLRHWGIPLSRRFRALKLWFVIRSYGVTGLQAYIRKHIKLAKLFETYVKNDARFEVSAPVNMGLVCFRLKGPNSLTKKLNRLINEAGQLHMVPALINKNYVIRFALCAENANENDIEFAWKAISAIASTLLARPSVEKSVSEYRASESDEEERSEETSEEDEEGDNAFMEFDNDIIFDESSTRMRSVMFRRSTFQRMISDPKCYDPKARGFSRGGRRRYMSESCSKRNPLSFDEPIFNF |
| DmDdc  (NP_724163.1) | MSHIPISNTIPTKQTDGNGKANISPDKLDPKVSIDMEAPEFKDFAKTMVDFIAEYLENIRERRVLPEVKPGYLKPLIPDAAPEKPEKWQDVMQDIERVIMPGVTHWHSPKFHAYFPTANSYPAIVADMLSGAIACIGFTWIASPACTELEVVMMDWLGKMLELPAEFLACSGGKGGGVIQGTASESTLVALLGAKAKKLKEVKELHPEWDEHTILGKLVGYCSDQAHSSVERAGLLGGVKLRSVQSENHRMRGAALEKAIEQDVAEGLIPFYAVVTLGTTNSCAFDYLDECGPVGNKHNLWIHVDAAYAGSAFICPEYRHLMKGIESADSFNFNPHKWMLVNFDCSAMWLKDPSWVVNAFNVDPLYLKHDMQGSAPDYRHWQIPLGRRFRALKLWFVLRLYGVENLQAHIRRHCNFAKQFGDLCVADSRFELAAEINMGLVCFRLKGSNERNEALLKRINGRGHIHLVPAKIKDVYFLRMAICSRFTQSEDMEYSWKEVSAAADEMEQEQ |
